# Dynamics of Active SiO_2_–Pt Janus Colloids in Dilute Poly(ethylene oxide) Solutions

**DOI:** 10.1021/acsphyschemau.2c00056

**Published:** 2023-01-25

**Authors:** Harishwar Raman, Sneham Das, Hrithik Sharma, Karnika Singh, Shruti Gupta, Rahul Mangal

**Affiliations:** † Department of Chemical Engineering, Indian Institute of Technology Kanpur, Kanpur208016, India; ‡ Department of Chemical Engineering, Jadavpur University, Kolkata700032, India

**Keywords:** Self-propelled Janus colloids, Self-diffusiophoresis, Active colloids, Poly(ethylene oxide), Polymer adsorption, Artificial micromotors, Janus colloids

## Abstract

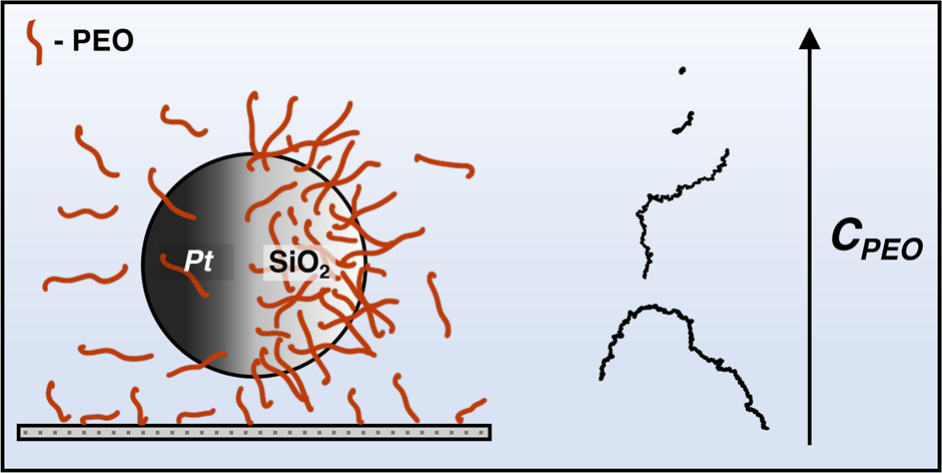

Self-propelled Janus colloids (JCs) have recently gained much attention due to their ability to move autonomously and mimic biological microswimmers. This ability makes them suitable for prospective drug/cargo-delivery applications in microscopic domains. Understanding their dynamics in surroundings doped with macromolecules such as polymers is crucial, as most of the target application media are complex in nature. In this study, we investigate the self-diffusiophoretic motion of hydrogen peroxide-fuelled SiO_2_–Pt JCs in the presence of dilute amounts of poly(ethylene oxide) (PEO). Despite the addition of PEO chains producing a Newtonian behavior with negligible increase in viscosity, the ballistic movement and rotational fluctuations of active JCs are observed to be significantly suppressed. With an increase in the polymer concentration, this leads to a transition from smooth to jittery to cage-hopping to the arrested motion of active JCs. We further propose that the anisotropic interaction of the polymers with the JC increases the “local drag” of the medium, resulting in the unusual impediment of the active motion.

## Introduction

Garnering inspiration from micro-organisms such as bacteria, algae, and spermatozoa, over the past couple of decades, researchers have devised *artificial micro-swimmers*.^1−4^ By utilizing an in-built mechanism which breaks the symmetry of their interactions with the surrounding medium, these artificial machines can perform autonomous motion analogous to the locomotion of microorganisms. On choosing appropriate chemistry, they typically self-generate either a chemical or a physical gradient in their vicinity, which drives their motion. Anderson first described this mechanism as *phoresis*.^5^ This unique ability to generate motion on micron length scales which overcomes the issue of Brownian motion, has intrigued researchers to control their motion and engineer them for targeted drug transport and delivery^6,7^ and environmental remediation^8^ and also to understand the collective dynamics of microbial colonies.^1,9,10^

A subset of artificial swimmers is *self-propelled colloids* which typically consist of Janus colloids (JCs)^11^ as the building block. Most commonly used active JCs are made of a silica or polystyrene (PS) core whose one-half is coated with platinum (Pt), which catalyzes the decomposition of hydrogen peroxide (H_2_O_2_) in an aqueous medium.^12^ The resultant asymmetric concentration gradient of the reaction products causes the JCs to propel in the direction away from the metal through a mechanism known as *self-diffusiophoresis*.^13−15^ JCs have also been known to propel under a self-generated electric potential (*self-electrophoresis*)^16,17^ or a temperature gradient (*self-thermophoresis*).^18−20^ The other widely recognized class of artificial swimmers consist of dispersed droplets self-propelling in the continuous phase in the presence of surfactants. Here, spontaneously generated Marangoni stresses at the droplet interface lead to their propulsion.^3^ While the propulsion mechanisms mentioned above may differ among the different artificial swimmers, their characteristics remain the same: a directed ballistic motion at short time scales followed by a random motion at longer time scales.^12,21^ However, the translational and rotational diffusivities depend on various parameters such as swimmer size,^22^ the magnitude of the propulsion force^12,23^ and also the presence of solutes in the surrounding medium.^24−26^ Microswimmer–wall effects have also been known to affect the motion characteristics significantly.^27−29^ Self-propelled JCs have been studied by subjecting them to diversified environments such as externally imposed flow,^30−32^ topological obstacles,^33,34^ and fluid interfaces^14,35,36^ or placing them in a pool of passive tracers.^37−39^ Recent studies have also demonstrated tuning of the dynamics of active JCs by altering their surface morphologies.^40,41^

To harness the full potential of artificial swimmers as drug carriers and transporters in bodily environments, it becomes critical to understand their behavior in polymeric and viscoelastic media, as most of the biological fluids are viscoelastic in nature.^42^ For biological swimmers such as *Escherichia coli*, Patteson et al. demonstrated that the elasticity of the polymer solution resulted in an enhanced swimmer speed and reduced tumbling tendency.^43^ Very recently, for active droplets in a viscoelastic media, Dwivedi et al. demonstrated a Peclet-induced transition in the swimming mode^25^ and also deformation in their shapes.^44^ For phoretic colloids, numerous theoretical and computational studies have attempted to understand the behavior of self-propelled colloids in complex media.^45−49^ So far, light-activated JCs have been chiefly used to study the dynamics of active JCs in viscoelastic media experimentally.^19,23,50,51^ Gomez-Solano et al. demonstrated that the bulk viscoelastic stresses decouple the translational and rotational dynamics of the active JC.^51^ Narinder et al. showed that increasing the propulsion force resulted in persistent cycloid trajectories.^23^ In addition, the JCs are also known to slow down upon reaching close to a solid boundary due to the resultant viscoelastic stresses that arise when the fluid between the JC and the boundary is compressed.^52^

Despite all these noteworthy experimental observations reported on light-activated JCs, only Saad and Natale have attempted to experimentally explore the behavior of the self-diffusiophoretic motion of SiO_2_–Pt active Janus colloids in a few dilute and semidilute polymer solutions. The study concluded that the polymeric entanglements physically confine the JCs and restrict their motion. However, the active JCs eventually overcome these constraints through their activity.^53^ Despite the interesting observations, the study neither explored the transition motion in detail, nor explored the effect on the rotational motion/orientation of the JCs. Given the underlying complex behavior, we strongly believe that more experimental studies are needed to better understand the dynamics of self-diffusiophoretic active JCs in complex fluids. Therefore, in this work, we experimentally study the motion of H_2_O_2_ fuelled SiO_2_–Pt JCs in dilute poly(ethylene oxide) (PEO) solutions over a wide range of polymer concentrations and molecular weights.

## Materials and Methods

### Materials

SiO_2_ particles (diameter ∼5 μm) were purchased from Sigma-Aldrich. Polystyrene (PS) particles (Duke, diameter ∼2 μm) were procured from Thermo Fisher. Polymers of poly(ethylene oxide) (PEO) of *M*_*w*_ ∼ 100, 600, 1000, and 8000 kDa in powder form were purchased from Sigma-Aldrich. H_2_O_2_ (pH 1.8–2.2, 30 wt %) was purchased from Thermo Fisher. Water with a measured resistivity of 18.2 MΩ cm was obtained from an ultrapure water unit (Barnstead Smart2Pure, Thermo Scientific). All the materials were used as procured.

### Methods

#### Synthesis of Janus Colloids

Janus colloids (JCs) are prepared using the protocol suggested by Love et al.^54^ Briefly, a SiO_2_ particle water suspension of submonolayer concentration is drop-cast on an O_2_ plasma-cleaned (PDC 32 Cleaner, Harrick Plasma) glass slide. Plasma treatment turns the glass slide hydrophilic, ensuring proper spreading of the water layer and preventing the formation of multilayers of particles during the drop-casting process. The cast drop is subjected to slow water evaporation, leaving a monolayer of particles. Subsequently, 15 nm of platinum (Pt) is sputter coated on the particles using a DC Magnetron Sputter Coater (BT 150, Hindustan High Vacuum). The self-shadowing effect of the microspheres ensures that only the top hemisphere of the particle is coated with Pt producing the JCs.

#### Sample Preparation and Viscosity Measurement

PEO–water stock solution of known concentration ∼0.1 wt % was prepared by overnight stirring of the PEO powder in ultrapure water, which is further diluted to the desired concentrations. The zero-shear viscosity (η_*o*_) of the prepared polymer solutions was measured using a viscometer (ROTAVISC lo-vi, IKA) in a concentric cylinder setup (ELVAS-1, IKA). Since all the polymer solutions used in this study are in the dilute regime, i.e., C_*PEO*_ ≪ C*, their rheological response is Newtonian (see Figure S1 in the Supporting Information) with a constant η_*o*_;^55^ also, due to low C_*PEO*_ the bulk viscosity (η_o_) of different PEO solutions does not vary significantly. Table 1 describes the properties of different polymer solutions prepared for our experiments.

**Table 1:** Description of Properties of Different Polymer Solutions

*M*_*w*,*PEO*_ (kDa)	*C*_*PEO*_(mg L^–1^)	C* (mg L^–1^)	η_0_(mPa s)
water (without PEO)	0	–	0.91
100	50	7513	1.03
100	100	7513	1.04
100	200	7513	1.08
100	300	7513	1.14
100	400	7513	1.26
600	10	1908	1.00
600	20	1908	1.02
600	30	1908	1.14
600	40	1908	1.20
600	50	1908	1.44
1000	10	1291	1.02
1000	20	1291	1.03
1000	30	1291	1.15
1000	40	1291	1.20
1000	50	1291	1.56
8000	10	264	1.04
8000	20	264	1.08
8000	30	264	1.12
8000	40	264	1.17
8000	50	264	1.81

The JCs are dispersed in these dilute PEO solutions, following which a known volume of 30 wt % hydrogen peroxide solution is added to achieve the desired fuel concentration of 3 wt %.

#### Imaging and Particle Tracking

To visualize JCs’ motion, a custom-made optical cell was prepared by placing a poly(dimethylsiloxane) (PDMS) spacer of height 1.5 mm and diameter 8 mm on a cleaned glass substrate. Glass slides were sonicated for 15 min in a 70 vol % isopropyl alcohol solution, followed by blowing with N_2_ and treating with O_2_ plasma for 5 min. The particle solution (with polymer and fuel) is added to the cell cavity and then sealed with a glass coverslip from the top. Due to density gradients, the JCs settle to the bottom of the well, making the system quasi-2-D. Imaging was done using an inverted microscope (IX73, Olympus) with a 20× objective in bright-field mode. The microscope was coupled with a CMOS Camera (Oryx 10GigE, Teledyne FLIR) of resolution 2048 × 2048 pixel^2^. Videos were recorded for approximately 150 s at 20 Hz. The center of mass positions the particles [*X*(*t*), *Y*(*t*)] were obtained using the MOSAIC plugin of the image processing software Fiji,^56^ which uses an image-correlation-based approach for feature-point tracking in the laboratory frame.^57^

#### Rheological Experiments

Flow sweep experiments were performed using a rheometer (DHR-3, TA Instruments) equipped with a concentric cylinder assembly. The temperature was maintained at 25 °C using a Peltier Jacket setup.

## Results and Discussion

**Figure 1:**
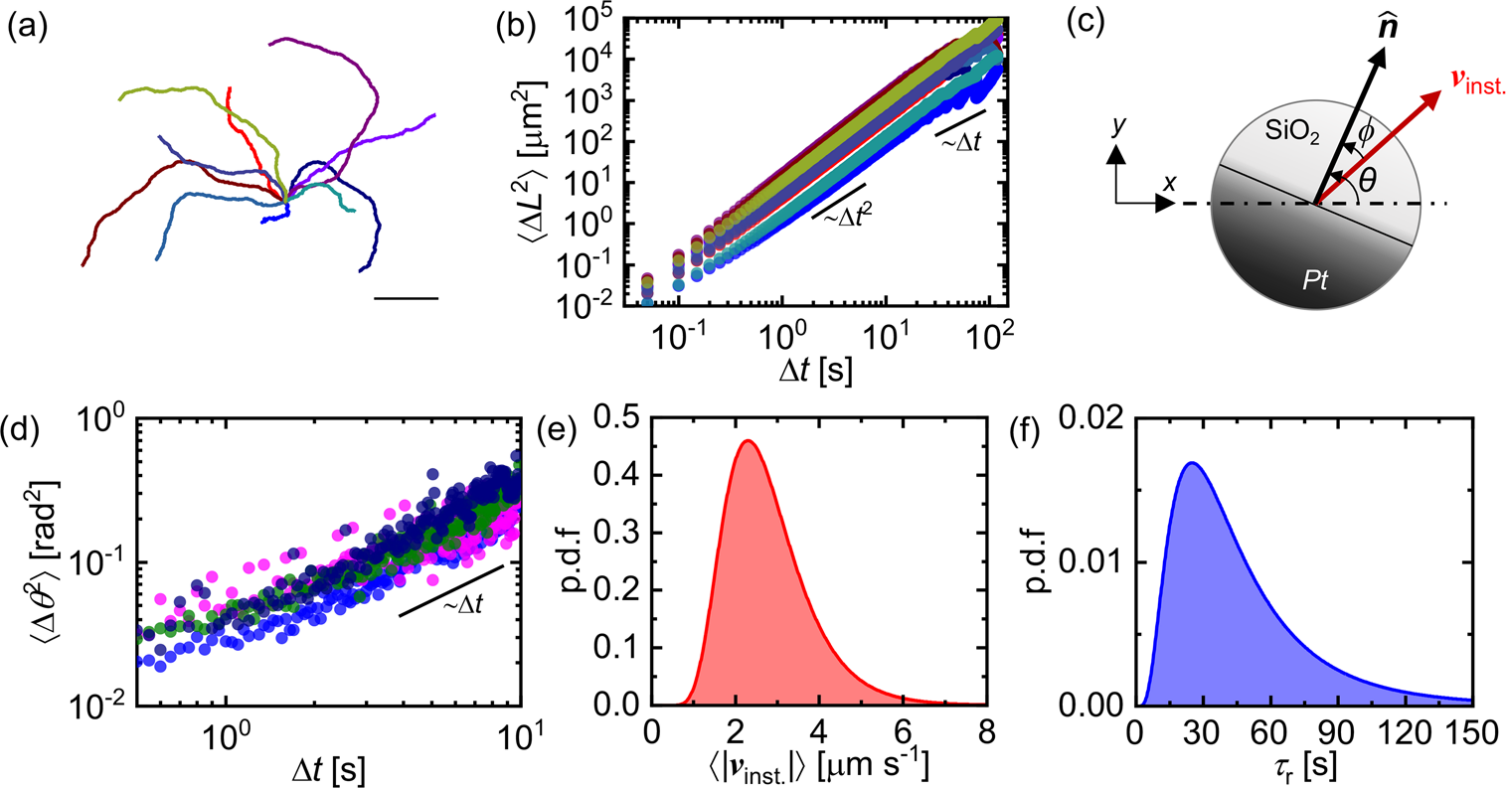
Dynamics of Janus colloids in the absence of PEO. (a) Representative 2-D trajectories (∼60 s) of active SiO_2_–Pt Janus colloids in 3 wt % H_2_O_2_ aqueous solution. The scale bar indicates a length of 50 μm. (b) Representative log–log plots of the 2-D MSDs corresponding to the trajectories shown in part a. (c) Schematic representation of an active JC indicating the orientation θ and the phase angle difference ϕ. (d) Representative log–log plots of the MSADs corresponding to selected trajectories in part a. (e,f) Probability distributions of average instantaneous speed ⟨|*v*_*inst*._|⟩ and reorientation time scale τ_*r*_ of nearly 20 active JCs. Note that, for better visualization, each of these curves is obtained by fitting a log-normal distribution to the histogram obtained for 20 active JCs.

By documenting the trajectories of around 20 JCs, we first report on the active motion of JCs in a 3 wt % H_2_O_2_ solution in the absence of any added polymer as our control experiment. A few representative trajectories, captured for ∼60 s, have been shown in Figure 1a. The absence of bulk convection in any direction is evident from the isotropic nature of the trajectories. Using these *X*–*Y* trajectories, we compute the two-dimensional mean square displacement (MSD) ⟨Δ*L*^2^⟩ of the JCs. A few representative MSDs are shown in Figure 1b. The active JCs perform directed motion at shorter time scales and subsequently transition to a random walk at longer time scales, indicated by the power-law slopes of 2 and 1, respectively. We also compute the mean square angular displacement (MSAD) ⟨Δθ^2^⟩ of the JCs using the orientation of the active JCs. As depicted in the schematic shown in Figure 1c, the orientation θ is defined as the angle between the unit normal vector to the equator of the JC and the *X*-axis in the *X*–*Y* plane. In Figure 1d, we show some representative MSAD curves of the active JCs in water. The power-law slope of 1 indicates the expected diffusive rotational behavior in the *X*–*Y* plane.

Parts e and f of Figure 1 show the probability distributions of the average instantaneous speed ⟨|*v*_*inst*._|⟩ and reorientation time scale τ_*r*_ respectively. The instantaneous speed ***v***_*inst***.**_ is defined as
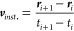
where **r**_**i**_(*X*(*t*_*i*_), *Y*(*t*_*i*_)) is the instantaneous position vector of the active JC. The reorientation time scale τ_*r*_ is defined as , where *D*_*R*_ is the rotational diffusion coefficient and is obtained by fitting the MSAD with the equation ⟨Δθ^2^⟩ = ω^2^Δ*t*^2^ + 2*D*_*R*_Δ*t*. Here, ω is the angular speed of the particles, which captures the cyclic nature of the JCs, if any. The variation in τ_*r*_ and ⟨|*v*_*inst*._|⟩ represents an inherent inhomogeneity in the dynamics of the active JCs which is expected to arise due to factors including nonuniform Pt coating and the heterogeneous interactions with the bottom wall.

**Figure 2:**
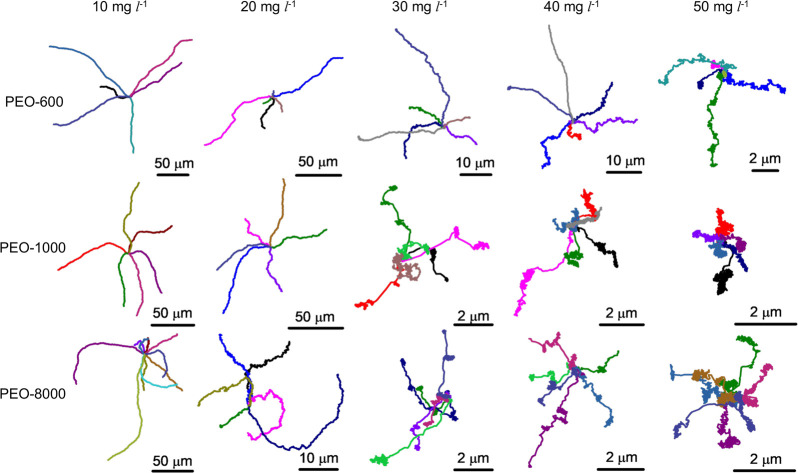
Representative trajectories (∼60 s) of 5 μm SiO_2_–Pt JCs in PEO solutions of molecular weights 600, 1000, and 8000 kDa with *C*_*PEO*_ up to 50 mg L^–1^, fuelled by 3 wt % H_2_O_2_.

After verifying the experimental setup and successfully performing the control experiments, we investigate the motion of the active JCs with PEO added to the surrounding media. From this point forward in the article, the PEO solutions will be denoted as PEO-*x*, where *x* denotes the molecular weight of PEO in kDa. Figure 2 shows few representative trajectories of JCs in PEO-600, PEO-1000 and PEO-8000 with varying PEO concentrations (*C*_*PEO*_) from 10 mg L^–1^ to 50 mg L^–1^. It is evident that for all PEO solutions, the trajectories become shorter with an increase in *C*_*PEO*_, which is accompanied by a transition from an almost smooth active motion, observed in just water (see Movie S1 in the Supporting Information), to a rather jittery motion with significant direction fluctuations at short time (see Movie S2 in the Supporting Information). The jitteriness in the motion for all three *M*_*w*_s for *C*_*PEO*_ = 30 and 40 mg L^–1^ was accompanied by an unusual stop-and-run type of active motion, wherein the active JCs move while stopping intermittently (see Movie S3 in the Supporting Information). The overall motion appears analogous to the bacterial run-and-tumble motion. With further increase of *C*_*PEO*_ to 50 mg L^–1^, the motion of active JCs was observed to be mostly arrested with negligible directed propulsion (see Movie S4 in the Supporting Information). Increasing *C*_*PEO*_ further (>50 mg L^–1^) did not result in any noticeable change. These observations indicate a PEO-induced hindrance in the motion of the active JCs.

**Figure 3:**
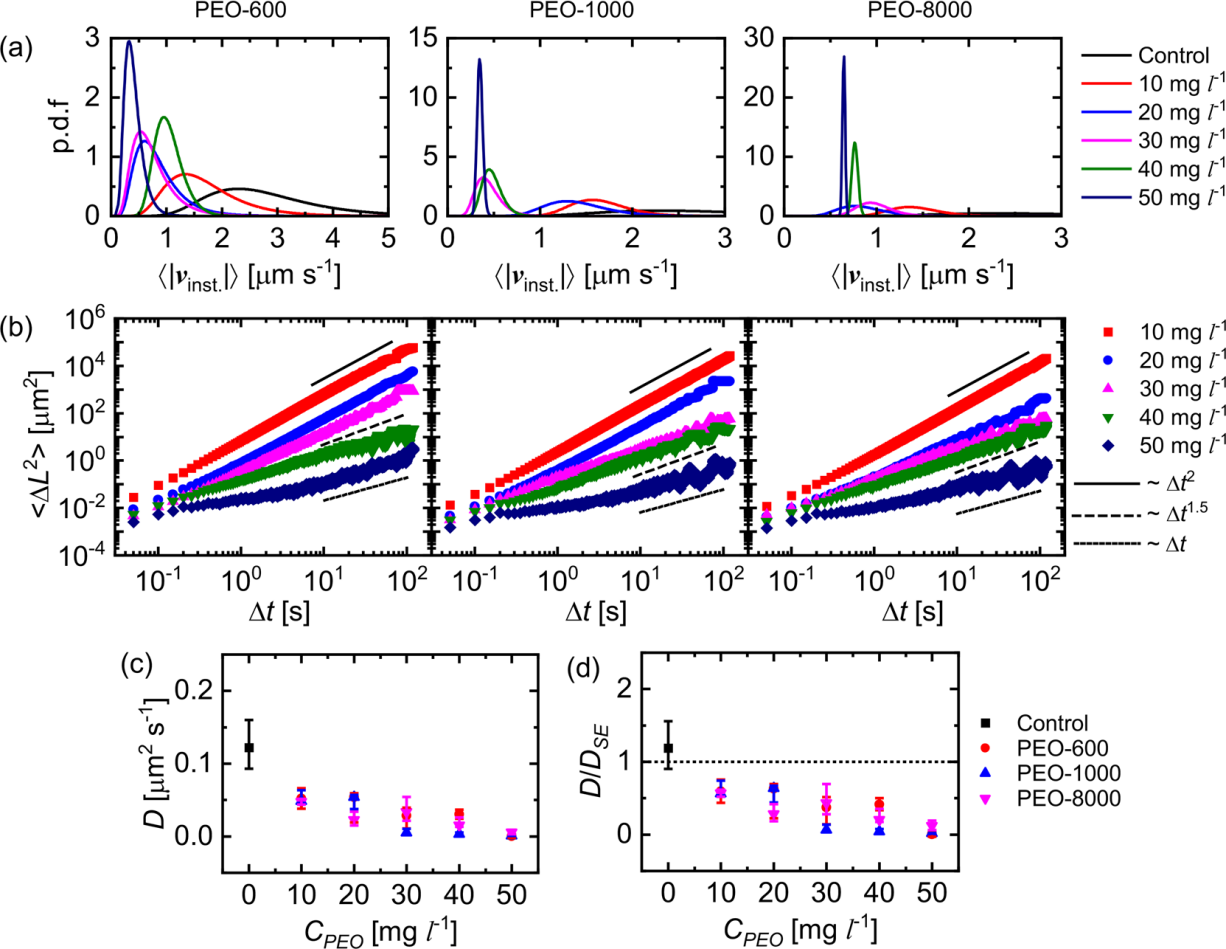
For active JCs in PEO solutions with *M*_*w*_ ∼ 600, 1000, and 8000 kDa. (a) Probability distribution curves of average speed ⟨|*v*_*inst*._|⟩. Note that, for better visualization, each of these curves is obtained by fitting a log-normal distribution to the histogram obtained for 20 active JCs. (b) Representative log–log plots of the 2-D MSDs with time. (c) Experimentally obtained diffusion coefficients *D* of active JCs in the PEO solutions. (d) Comparison of *D* with the Stokes–Einstein predictions in respective bulk media. Values reported are the medians, with the error bars corresponding to the first quartiles.

From the trajectories, ⟨|*v*_*inst*._|⟩ and the MSDs of the active JCs in PEO solutions are computed. The resulting ⟨|*v*_*inst*._|⟩ distributions are fitted with suitable log-normal distribution curves, shown in Figure 3a. Consistent with slower speed and more fluctuating motion with increasing *C*_*PEO*_, the values of ⟨|*v*_*inst*._|⟩ decrease. Since all the PEO solutions have similar η_*o*_, these observations contradict the theoretical formulation of phoretic speed proposed by Anderson .^5^Figure 3b shows a few representative MSD curves of the JCs in PEO solutions for the trajectories shown in Figure 2. With the increase in *C*_*PEO*_, the MSD curves show a decrease in long time-scale power-law slopes from 2 to 1.5 to 1, indicating a transition of motion from ballistic to hindered to random. This is consistent with the transition of trajectories from smooth to jittery to stop-and-run to arrested. This indicates that the increase in *C*_*PEO*_ significantly impedes the motion of the active JCs, wherein they are mostly trapped at moderate to long time scales. However, the short-time MSD slope of 2 at all PEO concentrations studied confirms the active nature of the JCs, which is further validated by their orientation vector aligning parallel to the *X*–*Y* plane,^58^ resulting in a half-moon conformation (see Figure S2 in the Supporting Information). We also obtain the diffusion coefficient *D* by fitting the MSD data with the equation
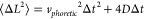
*v*_*phoretic*_ being the phoretic propulsion speed of the active JCs. The obtained values of *D* are shown in Figure 3c. For the control experiment, the median value of *D* is obtained as 0.122 μm^2^ s^–1^, nearly similar to the theoretical (Stokes–Einstein) prediction for a passive particle,
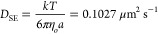


With increasing *C*_*PEO*_, the values of *D* decrease significantly. The comparison of the experimentally obtained *D* values with the Stokes–Einstein predictions in the different PEO solutions shown in Figure 3d indicate that the measured *D* values are lesser than the theoretical prediction even for the lowest polymer concentration, and this deviation is more pronounced for the highest polymer concentrations, where , despite the JCs being active. This further validates the polymer-induced hindered motion of the active JCs.

**Figure 4:**
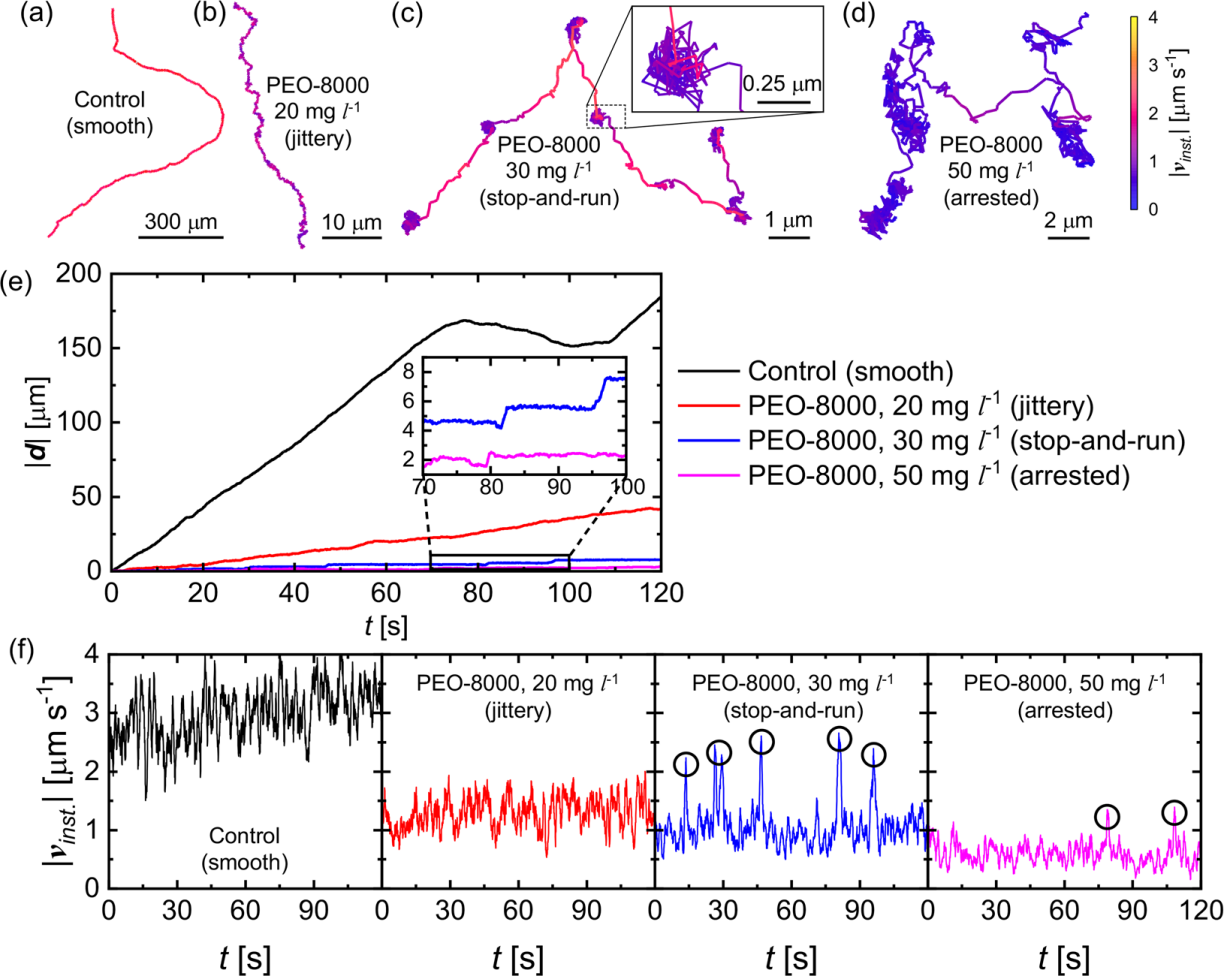
(a–d) Representative trajectories (∼120 s) illustrating the jitteriness and stop-and-run motion performed by a 5 μm SiO_2_–Pt JC in various PEO solutions. Color coding corresponds to the instantaneous speed |**v**_*inst*._|. Inset of part c shows a closer look at the tumbles during the stopped state. (e, f) Evolution of the magnitude of displacement |**d**| and the instantaneous speed |**v**_*inst*._| for the respective trajectories. Black circles in part f highlight the sudden spikes of |**v**_*inst*._|.

To further elaborate on the polymer-induced hindered motion of the active JCs, using the representative trajectories shown in Figure 4a–d, we calculate the transient evolution of the magnitude of the displacement vector |**d**| (=|**r**(*t*) – **r**(0)|) shown in Figure 4e. For smoother trajectories, |**d**| steadily increases with time. For jittery trajectories, |**d**| significantly drops and exhibits more fluctuations due to the frequent short-term direction changes. For stop-and-run type trajectories, |**d**| shows a step-type growth as shown in the inset. Here, the flatter sections indicate a stopped state and the sudden jumps correspond to the running events. For the arrested state, the steps are less apparent but still present for some active JCs. Next, Figure 4f shows the variation of |**v**_*inst*._| for trajectories shown in Figure 4a–d. As expected, with an increase in *C*_*PEO*_, the speeds of the active JCs are suppressed. Interestingly, for stop-and-run and arrested trajectories, sudden spikes in |**v**_*inst*._| are observed. As anticipated, the time instances at which these spikes are observed coincide with the timestamps when the step-type growth in |**d**| is observed, as shown in Figure 4e.

**Figure 5:**
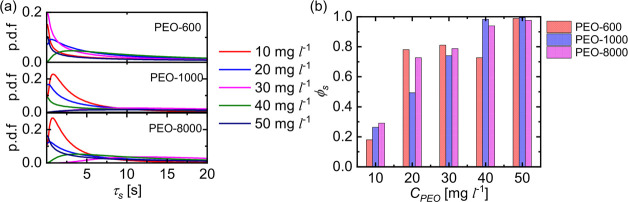
(a) Probability distribution of the stop time τ_*s*_ of the active JCs in PEO solutions of various concentrations. Note that, for better visualization, each of these curves is obtained by fitting a log-normal distribution to the histogram obtained for 20 active JCs. (b) Variation of stop fraction ϕ_*s*_ of the active JCs with PEO concentration for different *M*_*w*_s.

We also define the stop time τ_*s*_ as the duration for which the active JCs remain halted. It is calculated using the width of the plateau segments of the |**d**| *vs t* curve. Figure 5a shows the resulting probability distribution of τ_*s*_ for around 20 particles, each observed for 120 s. For all the molecular weights of PEO, the distribution of τ_*s*_ is observed to broaden as *C*_*PEO*_ increases. This is also demonstrated by the increase in the fraction of the total stop time ϕ_*s*_ with the increase in *C*_*PEO*_ (Figure 5b), which eventually saturates to 1 for the fully arrested motion.

**Figure 6:**
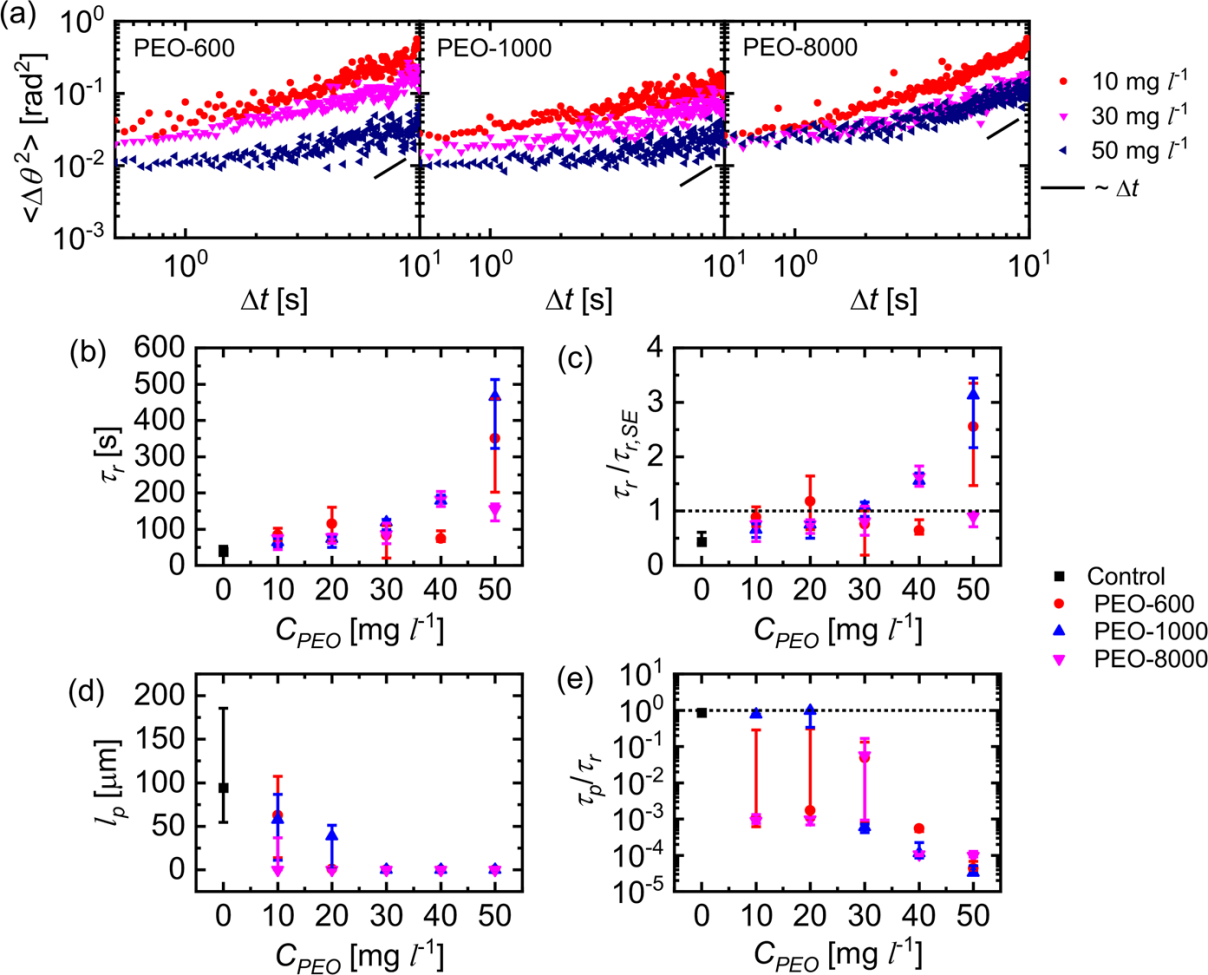
(a) Representative log–log plots of the MSADs of active JCs in different PEO solutions. (b) Experimentally obtained values of the reorientation time scale τ_*r*_ in different PEO solutions. (c) Comparison of the obtained τ_*r*_ values with the Stokes–Einstein values in respective solutions. (d) Experimentally obtained values of the persistence length *l*_*p*_ for different PEO solutions. (e) Comparison of the persistence time scale τ_*p*_ and the rotational time scale τ_*r*_ for different PEO solutions. Values reported in parts b–e are the medians, with the error bars corresponding to the first quartiles.

After analyzing the effect of PEO addition on the translation of the active JCs, we examine its effect on their rotation. In Figure 6a, we show a few MSAD curves of active JCs in various PEO solutions. We find that *D*_*R*_ reduces with an increase in *C*_*PEO*_, and hence, the reorientation time scale τ_*r*_ increases as shown in Figure 6b. The comparison of the obtained τ_*r*_ values with the Stokes–Einstein prediction (τ_*r*, SE_) shown in Figure 6c illustrates that with increasing *C*_*PEO*_, the active JCs undergo suppressed rotational events. These higher τ_*r*_ values suggest a more persistent translational motion, which is not in agreement with the nature of the trajectories shown earlier in Figure 2. To further understand this, we use the *X*–*Y* data to compute the persistence time scale τ_*p*_ from the unnormalized velocity autocorrelation function *C*(Δ*t*) defined as

**Figure 7:**
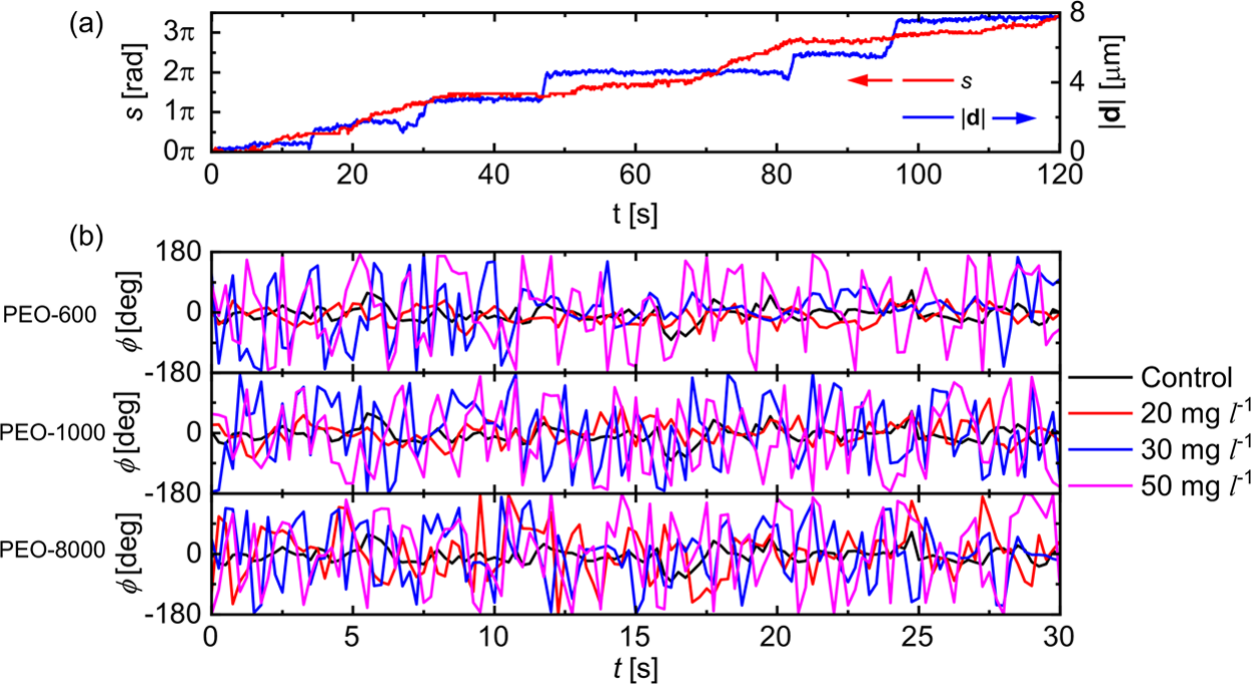
(a) Comparison of the translational and angular displacements for an active JC in 30 mg L^–1^ PEO-8000 solution. The corresponding trajectory is shown in Figure 4c. (b) Variation of the phase angle difference ϕ of the JCs with time, in different PEO solutions.

*C*(Δ*t*) = ⟨**v**_*inst*._(Δ*t*).**v**_*inst*._(0)⟩ and can be fitted using the equation
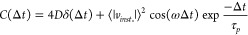
(see Supporting Information, Figures S3 and S4 for the representative *C*(Δ*t*) curves and the τ_*p*_ probability distribution). Figure 6d shows the variation of the persistence length *l*_*p*_(= ⟨|*v*_*inst*._|⟩τ_*p*_) with increasing *C*_*PEO*_. As expected, *l*_*p*_ decreases with an increase in *C*_*PEO*_, eventually approaching zero for stop-and-go and arrested-type motions. In Figure 6e, we compare the values of τ_*p*_ and τ_*r*_. For the control system (*C*_*PEO*_ = 0 mg L^–1^), the  values lie closer to 1 and decrease as *C*_*PEO*_ increases. At *C*_*PEO*_ = 50 mg L^–1^, the τ_*p*_ values are roughly 4 orders of magnitude lesser than τ_*r*_. This suggests that the velocity fluctuations, obtained using the JCs’ center of mass (*X*,*Y*) positions, are not correlated to their orientation fluctuations. Therefore, in the presence of PEO, the translation and the rotational motions of the active JCs are decoupled. To further validate this, we look into the transient dynamics of the active JCs. First, in Figure 7a, we compare the time evolution of |**d**| and the total angular displacement *s* for an active JC in 30 mg L^–1^ of PEO-8000 solution. Similar to |**d**|, we observe that *s* displays intermittent plateaus. However, the timestamps and the durations of these plateaus do not match that of |**d**|, suggesting that both the plateau events occur independently, indicating the decoupling of translation and rotation of the JCs. In addition, in Figure 7b, we plot the phase difference ϕ between the instantaneous velocity vector **v**_*inst*._ and the unit normal vector **n** for few active JCs. For the control case, ϕ values fluctuate close to 0, indicating an expected quasi-instantaneous response. However, with the increase in *C*_*PEO*_, the extent of fluctuations increases significantly, and we observe that they mostly originate from the fluctuations in **v**_*inst*._ (see Movies S1–S4 in Supporting Information). These enhanced fluctuations correspond to decreasing correlation between the translation and rotation motion of the active JCs.

Our observations of PEO-induced hindered translation motion of the active JCs are similar to a previous experimental report by Saad and Natale, who observed a decreased MSD slope of diffusiophoretic JCs in viscoelastic bulk media.^53^ Similarly, the decoupling of the translation motion and the orientation of the active JCs has been reported previously for active JCs in viscoelastic media.^23,51,59^ In these studies, the deviation in the observations for the active JCs compared to their motion in Newtonian media was attributed to the elasticity of the bulk medium. Also, Gomez-Solano et al. and Narinder et al. reported that for a given propulsion speed and the viscoelasticity of the surrounding medium, ϕ remains nearly constant,^23,51^ whereas, in our experiments, we observe that ϕ fluctuates randomly. Also, since the polymer solutions are in the Newtonian regime, our observations cannot be explained by the elastic effects of the surrounding bulk media. However, the similarity of the observations highlights the presence of a local random viscoelastic behavior near the JCs, caused by the presence of PEO chains.

The computational study of Theeyancheri et al. predicted a decrease in the MSD and MSAD slopes for Janus probes propelling with their sticky side forward in a medium containing polymers with sticky monomers.^59^ Since adsorption of PEO on SiO_2_ is well established,^60,61^ we expect that the sticky interactions between the SiO_2_ hemisphere of the active JC and the surrounding PEO chains may result in an increased concentration of polymer chains in the vicinity of the JC, thereby introducing a local viscoelastic response in the medium surrounding the particle. As the concentration of PEO in the bulk medium increases, the probability and hence the amount of PEO adsorbed on the silica surface is also expected to increase. In addition, we also expect the PEO chains to desorb, resulting in a dynamic adsorption–desorption process and generate randomness to the local viscoelastic stresses. Moreover, Rubio and Kitchener reported that an acidic medium, in virtue of its low pH, favors adsorption of PEO on SiO_2_.^60^ In our case, the measured pH values of all the aqueous H_2_O_2_ solutions (with and without PEO) lie in the range of 3.5–4.0, suggesting that our system favors PEO adsorption on the SiO_2_ hemisphere of the JCs. This adsorptive nature of PEO has been utilized in the preparation of shake gels containing silica nanoparticles and poly(ethylene glycol) (PEG).^62^ The adsorbed PEO can also screen the negative charges away from the SiO_2_ hemisphere, reducing the height of the active JC from the glass substrate. As a result, the local hydrodynamic drag increases, effectively lowering the translational diffusivity *D*, consistent with our results shown in Figure 3c.

Very recently, Banerjee et al. theoretically investigated the dynamics of artificial swimmers in compressible viscoelastic media that were modeled as passive tracers of a given volume fraction.^63^ The nature of the trajectories of the active JCs obtained in our experiments is qualitatively similar to their observations. This suggests that the effect of adsorbed PEO chains in our system can be conceived to generate local viscoelastic stresses similar to the passive tracers crowding the surrounding medium. However, the decreasing rotational diffusivities at higher *C*_*PEO*_ contrasts the recent studies reporting an enhanced rotational diffusivity of caged active Brownian colloids in glassy systems^37,64^ and light-activated JCs in viscoelastic polymer solutions.^51^ These studies attribute the enhanced rotation of active JCs to increased collisions and bulk viscoelastic stresses. Unlike these reports, in our work, the PEO adsorption-induced collisions/viscoelastic stresses are expected to be local and anisotropic, which may explain the differences.

**Figure 8:**
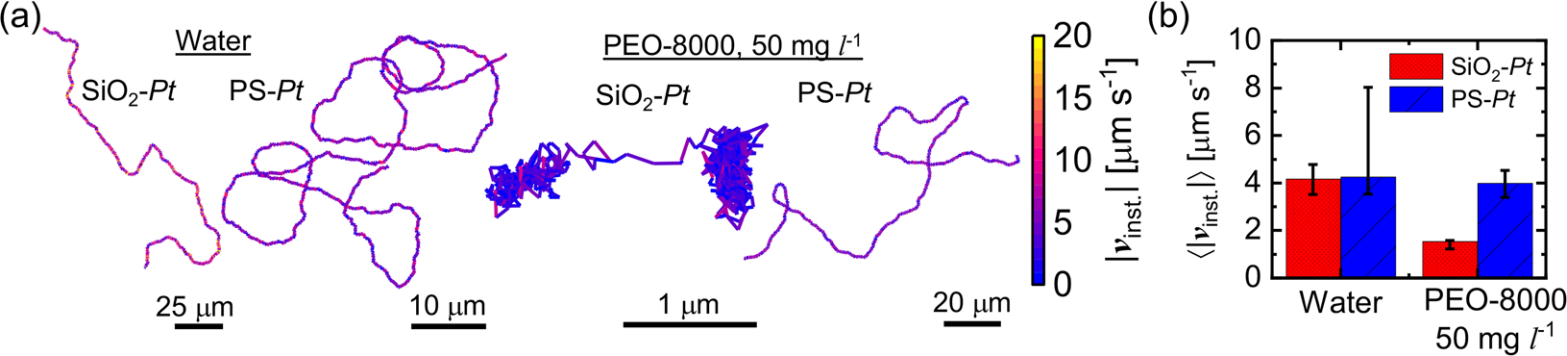
(a) Trajectories (∼60 s) of 2 μm SiO_2_–Pt and PS-Pt JCs in water and 50 mg L^–1^ PEO-8000 solutions. (b) Average instantaneous speeds ⟨|*v*_*inst*._|⟩ of the active JCs in water and 50 mg L^–1^ PEO-8000 solutions. Reported values are the medians with the error bars corresponding to the first quartiles.

To further validate our hypothesis and confirm the influence of the active JCs’ surface chemistry on the PEO-induced hindered motion, we also conduct additional experiments with 2 μm polystyrene–Pt (PS–Pt) active JCs and compare it with 2 μm SiO_2_–Pt active JCs, both in water and in PEO-8000 solution with *C*_*PEO*_ = 50 mg L^–1^. In Figure 8a, we show the representative trajectories of the 2 μm JCs in both solutions. We find that the SiO_2_–Pt JCs were arrested in the PEO-8000 solution. In contrast, the PS-Pt JCs show a comparatively significant movement, which is further verified by the comparison of their average instantaneous speeds ⟨|*v*_*inst*._|⟩ shown in Figure 8b. Although PEO is known to adsorb on polystyrene as well, unlike the SiO_2_–Pt JCs, the hydrogen bonding interactions with the silanol groups are absent,^65^ thereby allowing the PS-based JCs to move freely. While adsorbed on the SiO_2_–Pt active JCs, PEO chains can also adsorb on the silica molecules in the glass substrate. This, in turn, can increase the roughness of the substrate, enhancing the overall resistance to motion of the SiO_2_-based JCs. The resistance caused by polymer adsorption on the substrate could be eliminated by adjusting the substrate properties or choosing an appropriate density-matched bulk environment, enabling the active JC to stay away from the substrate, which is yet to be explored.

**Figure 9:**
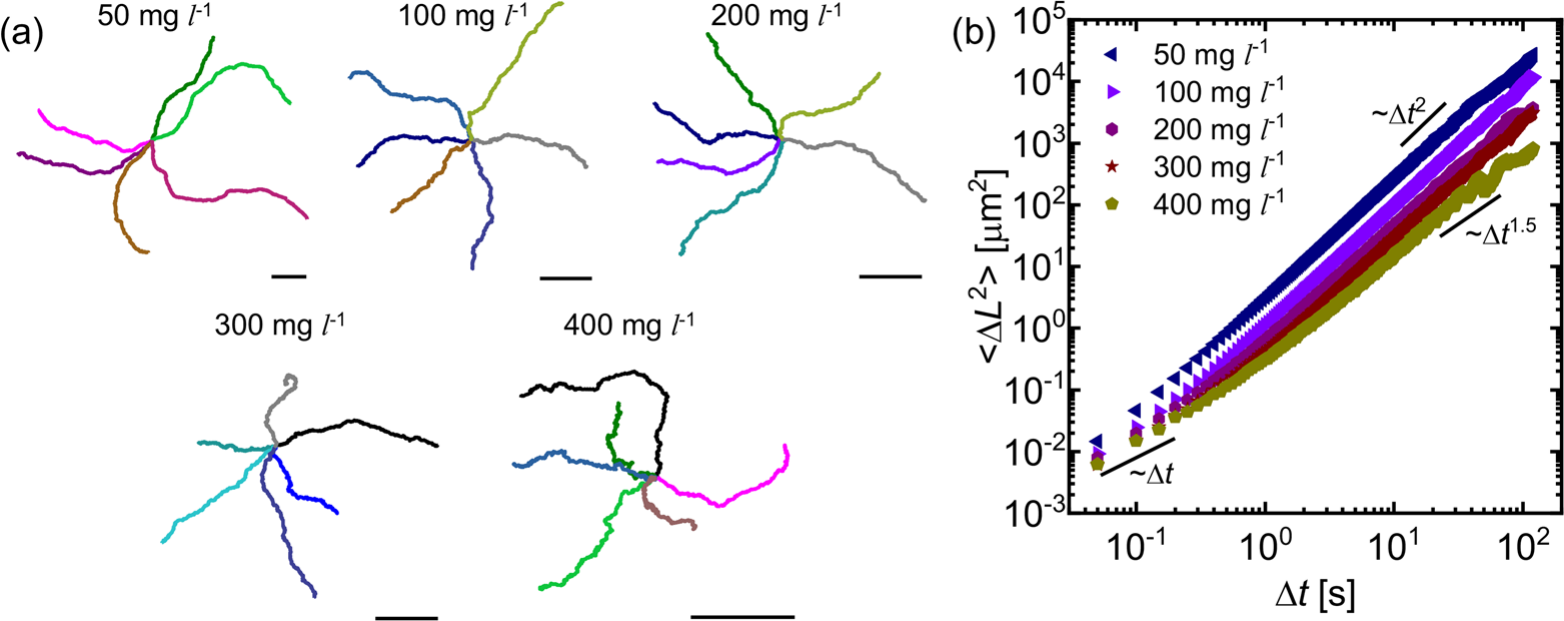
SiO_2_–Pt JCs in dilute PEO-100 solutions: (a) Representative trajectories (∼60 s) of the active colloids in PEO-100 solutions of concentrations up to 400 mg L^–1^. Scale bars indicate a length of 20 μm. (b) Representative 2-D MSD plots of the active JCs.

Given the interactions between the PEO chains, the SiO_2_–Pt active JCs and the glass substrate, we speculate the polymer adsorption-induced resistance to be less pronounced for shorter PEO chains i.e. lower *M*_*w*_. To confirm this, we perform additional experiments in PEO-100 solutions, with *C*_*PEO*_ ≥ 50 mg L^–1^. From the representative trajectories of active JCs shown in Figure 9a, the active JCs appear to exhibit better mobility when compared to those observed in earlier experiments with higher *M*_*w*_s of PEO, as predicted. Also, even for the PEO-100 solution containing *C*_*PEO*_ = 400 mg L^–1^ (≫the highest *C*_*PEO*_ used for other higher *M*_*w*_ PEO solutions), we only observe the onset of a jittery motion without any noticeable stop-and-run or arrested events. The MSD plots shown in Figure 9b slope down to 1.5 at *C*_*PEO*_ = 400 mg L^–1^, which is similar to the active JCs performing a jittery motion, as earlier seen in Figure 3b. As expected, the values of ⟨|**v**_*inst*._|⟩ and τ_*p*_ also decrease with an increase in the concentration of PEO-100 (see Figure S5 in the Supporting Information). While the PEO-100 solutions have a similar bulk viscosity (η_*o*_) as that of the other PEO solutions, clearly, in PEO-100 solutions, the active JCs experience significantly lesser resistance to motion. This confirms that the length of the polymer chain adsorbed on the active JCs also plays a key role in determining their retarding effect on the motion of the JCs.

## Summary

In conclusion, we present a detailed experimental study on the motion of H_2_O_2_ fuelled SiO_2_–Pt active JCs in dilute PEO aqueous solutions. For a given molecular weight of PEO, with increasing PEO concentration, we observe a transition of active JCs’ translational motion from smooth to jittery to stop-and-run and eventually to an arrested state. In addition, the rotation of the active JCs is also suppressed with an increase in polymer concentration. Despite the polymer in the surroundings being in the dilute concentration regime, the extent of suppression in the motion of the active JCs is remarkable. Our experiments indicate that this transition is triggered by the preferential adsorption of PEO on the SiO_2_ surface of the JC and also on the glass substrate. This anisotropic adsorption of PEO renders viscoelastic properties to the local medium surrounding the JC, generating random stresses hindering their active motion. The polymer adsorption-induced retardation of the active JCs is shown to be dictated by the polymer chain length and the concentration. In addition, we speculate that modifying the surface morphologies of the active JCs or grafting them with polymers of different chain lengths might also lead to exciting results and a better understanding of the underlying mechanism behind our experiments, which we intend to explore in the future. As we have demonstrated that the physicochemical properties of both the artificial swimmer and the surrounding medium significantly influence the swimmer’s motion, our study provides valuable insights into engineering active Brownian colloids for targeted delivery applications in various bodily fluids.

## Data Availability

The data that support the findings of this study are available from the corresponding author upon reasonable request.

## Supporting Information Available

Movie S1: Active JCs in water (without polymer) (MP4)
Movie S2: JC performing jittery motion (PEO-8000, 20 mg L^–1^) (MP4)
Movie S3: JC performing stop-and-run type movement (PEO-8000, 30 mg L^–1^) (MP4)
Movie S4: JC in locally arrested state (PEO-8000, 50 mg L^–1^) (MP4)
Flow sweep curves of PEO solutions containing 50 mg L^–1^ of PEO, optical micrographs of active JCs in 50 mg L^–1^ polymer solutions, velocity autocorrelation function curves of JCs in different polymer solutions, probability distribution of the persistence time in different polymer solutions, and dynamics of JCs in PEO-100 solutions (PDF)
