# Supplementary material for: Dynamics of Active SiO2–Pt Janus Colloids in Dilute Poly(ethylene oxide) Solutions
Source: ACS Phys Chem Au. 2023 Jan 25;3(3):279–89. doi: 10.1021/acsphyschemau.2c00056 (PMC10214528; doi:10.1021/acsphyschemau.2c00056)
Supplement: Supplementary file 5 — pg2c00056_si_005.pdf [file pg2c00056_si_005.pdf]

Supporting Information

Dynamics of active SiO_2-Pt Janus Colloids in dilute Poly(Ethylene Oxide) solutions

Harishwar Raman,† Sneham Das,‡ Hrithik Sharma,† Karnika Singh,† Shruti Gupta,† and Rahul Mangal*,†

†*Department of Chemical Engineering, Indian Institute of Technology Kanpur, Kanpur, India.*

‡*Department of Chemical Engineering, Jadavpur University, Kolkata, India.*

E-mail: mangalr@iitk.ac.in

List of Movies

S1: Active JCs in Water (without polymer)

S2: JC performing jittery motion (PEO-8000, 20 mg l^{-1})

S3: JC performing stop-and-run type movement (PEO-8000, 30 mg l^{-1})

S4: JC in locally arrested state (PEO-8000, 50 mg l^{-1})

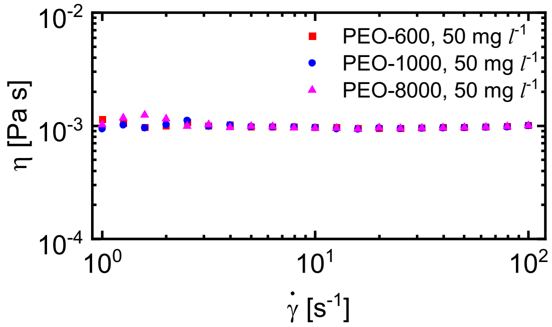

Figure S1: Viscosity as a function of shear rate for PEO solutions containing 50 mg l^{-1} of PEO.

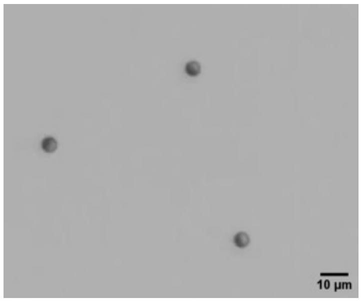

Figure S2: Optical Micrograph showing the active JCs in 50 mg l^{-1} PEO-8000 solution.

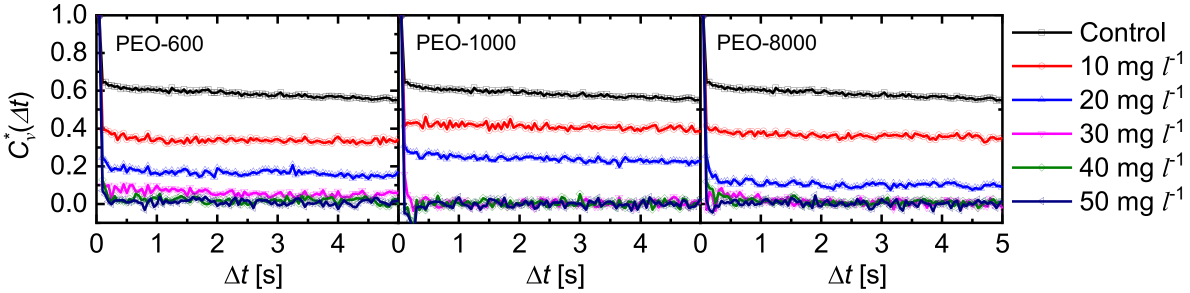

Figure S3: Representative normalized Velocity Autocorrelation Function curves of JCs in different polymer solutions.

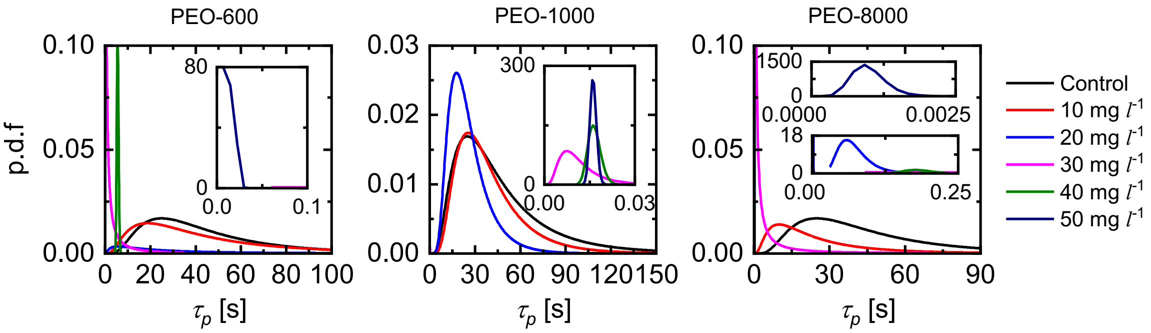

Figure S4: Probability distributions of τ_p for JCs in different PEO solutions. Note that for better visualization, each of these curves is obtained by fitting a log-normal distribution to the histogram obtained for 20 active JCs.

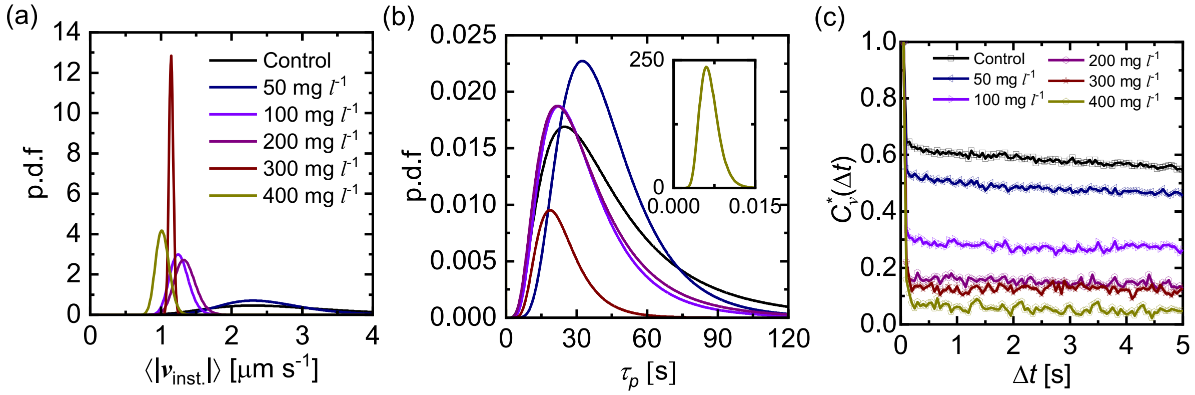

Figure S5: (a, b) Probability distributions of $\langle|v_{inst.}|\rangle$ and τ_p for JCs in different concentrations of PEO-100. Note that for better visualization, each of these curves is obtained by fitting a log-normal distribution to the histogram obtained for 20 active JCs. (c) Representative normalized Velocity Autocorrelation Function curves of JCs in PEO-100 solutions.
